# PD-L1 expression in pleomorphic, spindle cell and giant cell carcinoma of the lung is related to TTF-1, p40 expression and might indicate a worse prognosis

**DOI:** 10.1371/journal.pone.0180346

**Published:** 2017-07-03

**Authors:** Violaine Yvorel, Arnaud Patoir, François Casteillo, Claire Tissot, Pierre Fournel, Marie-Laure Stachowicz, Georgia Karpathiou, Olivier Tiffet, Michel Péoc’h, Fabien Forest

**Affiliations:** 1 Department of Pathology, North Hospital, University Hospital of Saint Etienne, Saint Etienne, France; 2 Department of Thoracic Surgery, North Hospital, University Hospital of Saint Etienne, Saint Etienne, France; 3 Department of Pneumology, North Hospital, University Hospital of Saint Etienne, Saint Etienne, France; 4 Department of Oncology, Lucien Neuwirth Cancer Institute, Saint Priest en Jarez, France; University of South Alabama Mitchell Cancer Institute, UNITED STATES

## Abstract

Lung sarcomatoid carcinoma of the lung is a rare tumor with a poor prognosis. More than 90% of them are pleomorphic, spindle cell and giant cell carcinoma (PSCGCC). This rare subtype of lung cancer is thought to be more resistant to chemotherapy, and a small subset of them seems to exhibit targetable mutations. Immunotherapy against PD1/PDL-1 is a new emerging treatment, and might be of interest in PSGSCC because they frequently express PD-L1. The aim of our work is to evaluate PD1 and PDL-1 expression in a surgical series of lung PSCGCC and their relationship with morphological and immunohistochemical parameters and prognosis. Thirty-six patients who underwent surgical resection of a PSGSCC were included. PD-L1 (E1L3N) expression on tumor cells and PD1 (NAT105) expression by tumor infiltrating lymphocytes (TILs) were performed by immunohistochemistry. Results were compared to immunohistochemistry tests of TTF1, Napsin A, p40 and to molecular study of EGFR, KRAS, BRAF and HER2. Seventy-five % of PSCGCC were considered as positive for PD-L1.PD-L1 expression in PSGSCC is associated with TTF-1 and/or Napsin A expression (47.2%, p = 0.039). Few p40 positive PSCGCC expressed PD-L1 (8.3%, p = 0.013). PD1 expression was not related to TTF-1 and/or Napsin A expression (p = 0.47), p40 expression (p = 0.68) or survival (p = 0.14). PD-L1 or PD1 expression were not related to the age, gender, pT, pN, stage, visceral pleura invasion, histopathological subtype, the presence of giant cell component, the predominance of sarcomatoid component, and the presence of EGFR or BRAF or HER2 or PIK3CA mutation (p>0.05). PD-L1 expression was correlated with a worse overall survival in PSCGCC (p = 0.045). PD-L1 expression is frequent in PSCGCC and might be associated with the expression of adenocarcinoma markers (TTF-1, Napsin A) or the lack of expression of squamous cell carcinoma marker (p40).

## Introduction

Lung sarcomatoid carcinoma (SC) is a rare type of lung carcinoma with a poor prognosis, representing less than 1% of lung cancer. In the current 2015 WHO classification, 3 subtypes are recognized: pleomorphic, spindle cell, and giant cell carcinoma (PSCGCC), carcinosarcoma and pulmonary blastoma. More than 90% of SC is represented by PSCGCC. SC might be more resistant to chemotherapy and radiotherapy than other “non-small cell” lung carcinomas (NSCLC). We have recently showed that a subset of PSCGCC has a targetable mutation and might respond to targeted therapy [1]. Nevertheless, like other NSCLC, few PSCGCC have a targetable mutation. Recently, PD1/PD-L1 inhibitors were developed. For all the developed drugs, the consequence is an increased recognition of tumor cells by immune cells. In consequence, the survival is better, especially in patients positive for PD-L1 immunohistochemistry [2]. In NSCLC, PD-L1 expression is the highest in squamous cell carcinoma of smokers and is related to solid subtype and KRAS mutation in adenocarcinoma. In NSCLC, PD-L1 expression is found either higher in squamous cell carcinoma than in adenocarcinoma or higher in adenocarcinoma than in squamous cell carcinoma[10+ [ref D’inceco,]. A recent study has showed that PD-L1 expression is more frequent in SC than in NSCLC [3].

We previously suggested that the presence of molecular targets in PSCGCC might be related to their morphological or immunohistochemical differentiation [1]. Nevertheless, the relationship between PD-L1 and PD1 expression and morphological differentiation or immunohistochemical factors have not been studied in PSCGCC.

The aim of our work is to evaluate PD1 and PDL-1 expression in in a surgical series of PSCGCC and their relationship with their differentiation and prognosis.

## Material and methods

### Patients, histopathological data and statistical analysis

This study was conducted in the Pathology Unit of the Saint Etienne University Hospital. Authorization to use human biological samples for research was obtained (no. AC-2013-1835). Approval of the study by the local Ethical committee of CHU de Saint Etienne was obtained for the collection of paraffin embedded tissue samples for immunohistochemical testing. Each patient gave written consent for sample use for research.

All the consecutive patients who underwent a surgical resection of PSCGCC in our hospital were included. Thirty-six patients were identified from 1997 to 2016. The histopathological diagnosis was confirmed by two pathologists (VY, FF) according to the current 2015 WHO classification [4]. Patients were staged according to the current TNM staging (7^th^ Edition). Clinical data, histopathological data, p40 (polyclonal, dilution 1:75, Clinisciences, Nanterre France), Napsin A (clone IP64, dilution 1:400, Novocastra, Nanterre France) and TTF-1 (clone: 8G7G3/1, dilution 1:50, Dako, Courtaboeuf France) immunohistochemistry, and molecular data for most patients were taken from a previous publication of our group [1].

Categorical variables were compared by Fisher’s exact test. Overall survival (OS) was assessed using the Kaplan-Meier method, whereas a log-rank test used for comparisons was performed with R software for Linux (Version 3.2.3) with Rstudio for Linux (Version 1.0.136). All reported p values are two-sided and p < 0.05 was considered significant.

### PD1 and PD-L1 immunohistochemistry

To measure PD-L1 expression, we used a rabbit anti-B7-H1(PD-L1) monoclonal antibody (clone E1L3N, dilution = 1/200, Cell Signaling Technology, Danvers, MA) and a PD1 antibody (clone NAT105 dilution = 1/50, Abcam, Cambridge, UK). Automated immunohistochemistry (Bond III; Menarini Diagnostics, Rungis, France) was performed on 4-μm-thick sections of paraffin-embedded tissue blocks. The expression of PD-L1 and PD1 is heterogeneous in lung cancer so its quantification should be as exhaustive as possible. In consequence, immunochemistry against PD-L1 and PD1 was performed on full section instead of tissue micro-array which might not be appropriate for this study [5].

PD-L1 tumor positivity was defined as ≥5% tumor cell membrane staining like in other studies on PSCGCC [3,6]. The staining of immune cells by PD-L1 was not considered as a positive staining but only served as an internal positive control. The percentage of tumor cells stained was recorded.

PD1 positivity was defined by the presence of tumor infiltrating lymphocytes (TILs) stained in the tumor stroma. Immune cells stained outside the tumor stroma were considered as negative.

### Literature search

A comparison of our results with published results of PD-L1 in SC has been performed. A search through pubmed search engine has been performed on the 20^th^ of January 2017 with the following search terms: ("antigens, cd274"[MeSH Terms] OR ("antigens"[All Fields] AND "cd274"[All Fields]) OR "cd274 antigens"[All Fields] OR ("pd"[All Fields] AND "l1"[All Fields]) OR "pd l1"[All Fields]) AND ("carcinoma"[MeSH Terms] OR "carcinoma"[All Fields] OR ("sarcomatoid"[All Fields] AND "carcinoma"[All Fields]) OR "sarcomatoid carcinoma"[All Fields]) AND ("lung"[MeSH Terms] OR "lung"[All Fields])

## Results

### Intratumoral PD-L1 expression

**Fig 1:**
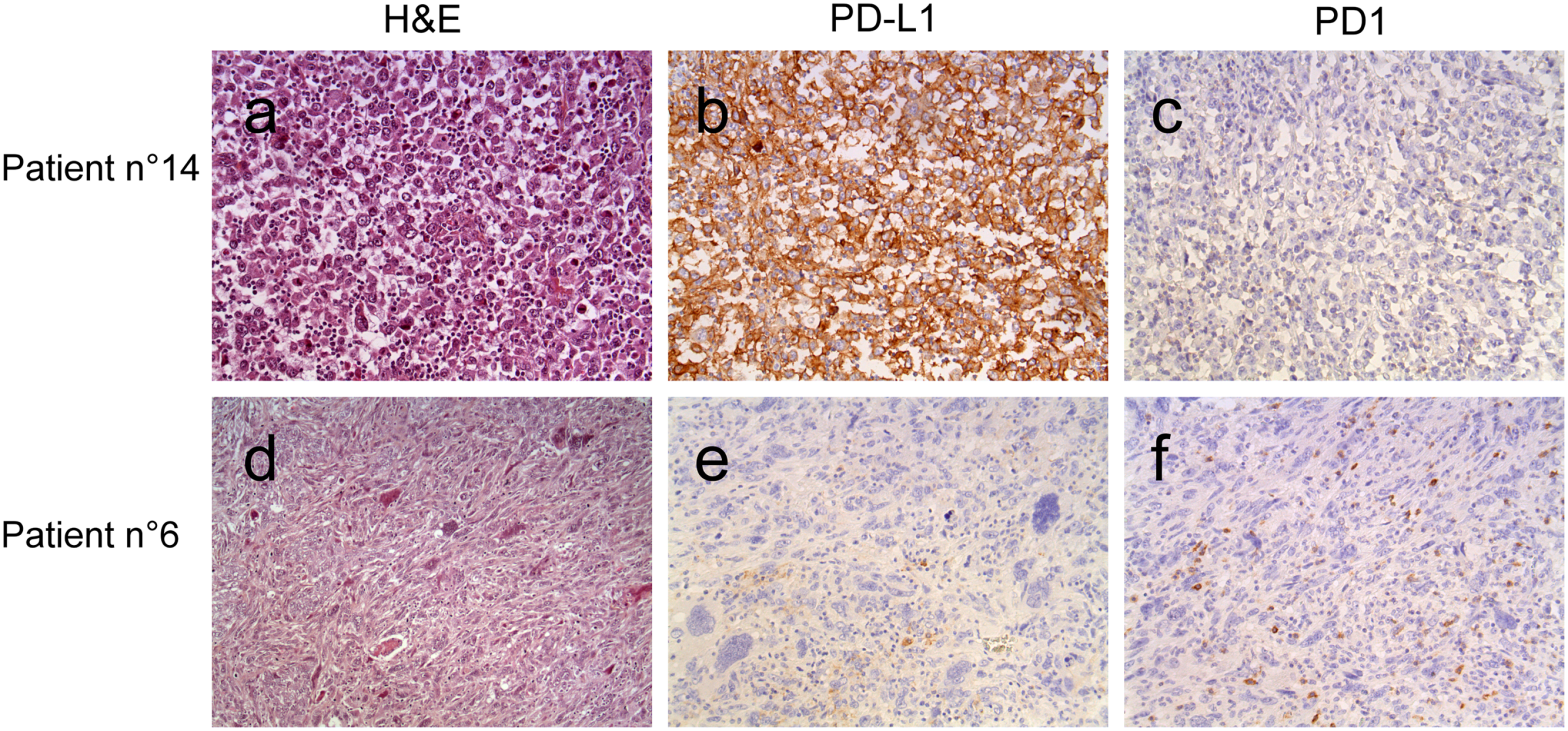
Microphotograph of 2 cases, at x200 magnification for H&E and immunohistochemistry. Patient 14 was frankly positive with rare PD1 positive TILs. Patient 6 was negative for PD-L1, rare immune cells stained serving as an internal control. PD1 positive TILs were present within the tumor.

PD-L1 was negative for 9 (25%) patients, and was found positive for 27 (75%) of patients (Fig 1). Sixteen of positive patients had an expression ≥50% of PD-L1. None of the tumors had an expression between 1% and 5%.

PD-L1 positivity was related to p40 expression (p = 0.013) and to TTF-1 and or Napsin expression (p = 0.039) in PSCGC carcinoma (Table 1). PD-L1 positivity was not related to the age (p = 0.69), gender (p = 65), pT (p = 0,13), pN (p = 1), stage (p = 0.26), visceral pleura invasion (p = 0.055), histopathological subtype (p = 1), the presence of giant cell component (p = 0.23), the predominance of sarcomatoid component (p = 0.27), and the presence of EGFR or BRAF or HER2 or PIK3CA mutation (p = 0.15) (Table 1).

**Table 1:** Clinical and pathological data related to PD-L1 and PD1 expression.

N = 36 (100%)	PD-L1- n (%)	PD-L1+ n (%)	p-value	PD1- n (%)	PD1+ n (%)	p-value
**Age**			0.69			0.47
≤60	2 (5,6)	9 (25)		3 (8,3)	8 (22,2)	
>60	7 (19,4)	18 (50)		11 (30,6)	14 (38,9)	
**Gender**			0.65			0,67
M	1 (2,8)	6 (16,7)		5 (13,9)	2 (5,6)	
F	8 (22,2)	21 (58,3)		16 (44,4)	13 (36,1)	
**pT**			0.13			0.51
T1	1 (2,8)	0 (0)		1 (2,8)	0 (0)	
T2	5 (13,9)	8 (22,2)		7 (19,4)	5 (13,9)	
T3	3 (8,3)	15 (41,7)		12 (33,4)	7 (19,4)	
T4	0 (0)	4 (11,1)		1 (2,8)	3 (8,3)	
**pN**			1			0,74
N0	5 (13,9)	14 (38,9)		8 (22,3)	11 (30,5)	
N1&N2	4 (11,1)	13 (36,1)		6 (16,7)	11 (30,5)	
**Stage**			0.26			0.95
I	3 (8,3)	2 (5,5)		2 (5,5)	3 (8,3)	
II	3 (8,3))	12 (33,4)		5 (13,9)	10 (27,8)	
III	3 (8,3)	11 (30,6)		6 (16,7)	8 (22,2)	
IV	0 (0)	2 (5,6)		1 (2,8)	1 (2,8)	
**Visceral pleura invasion**			0.055			1
PL0	7 (19,4)	10 (27,8)		10 (27,8)	7 (19,4)	
PL1, 2 &3	2 (5,6)	17 (47,2)		12 (3,4)	7 (19,4)	
**Histopathological subtype**			1			0.36
Pleomorphic carcinoma	8 (22,2)	23 (63,9)		11 (30,6)	20 (55,5)	
Other subtypes	1 (2,8)	4 (11,1)		3 (8,3)	2 (5,6)	
**TTF-1 and/or Napsin A immunohistochemistry**			**0.039**			0.47
Negative	0 (0)	10 (27,8)		11 (30,6)	14 (38,9)	
Positive	9 (25,0)	17 (47,2)		3 (8,3)	8 (22,2)	
**p40**			**0.013**			0.68
Negative	4 (11,1)	24 (66,7)		10 (27,8)	18 (50,0)	
Positive	5 (13,9)	3 (8,3)		4 (11,1)	4 (11,1)	
**Predominant component**			0.27			1
Sarcomatoid component	4 (11,1)	18 (50,0)		9 (25,0)	13 (36,1)	
Non-sarcomatoid component	5 (13,9)	9 (25,0)		5 (13,9)	9 (25,0)	
**Giant cell component**			0.23			0.48
Present	4 (11,1)	19 (52,8)		12 (33,3)	11 (30,6)	
Absent	5 (13,9)	8 (22,2)		9 (25,0)	4 (11,1)	
**EGFR or BRAF or HER2 or PIK3CA mutation**			0.15			0.39
Absent	7 (19,4)	16 (44,5)		8 (22,2)	15 (41,6)	
Present	0 (0)	7 (19,4)		1 (2,8)	6 (16,7)	
Impossible	2 (5,6)	4 (11,1)		5 (13,9)	1 (2,8)	

### Intratumoral PD1 expression

Eighteen patients had an expression of PD-L1 and PD1.Intra-tumoral PD1 expression was not related to PDL1 expression (p = 0.45). PD1 was positive in 22 patients and negative in 14 patients.

PD1 expression was not related to the age (p = 0.47), gender (p = 0.67), pT (p = 0,51), pN(p = p = 0.74), stage (p = 0.95), visceral pleura invasion (p = 1), histopathological subtype (p = 0.36), the presence of giant cell component (p = 0.48), the predominance of sarcomatoid component (p = 1),TTF-1 or Napsin A positivity (p = 0.47), p40 expression (p = 0.68) and the presence of EGFR or BRAF or HER2 or PIK3CA mutation (p = 0.39) (Table 1).

### Survival analyses

**Fig 2:**
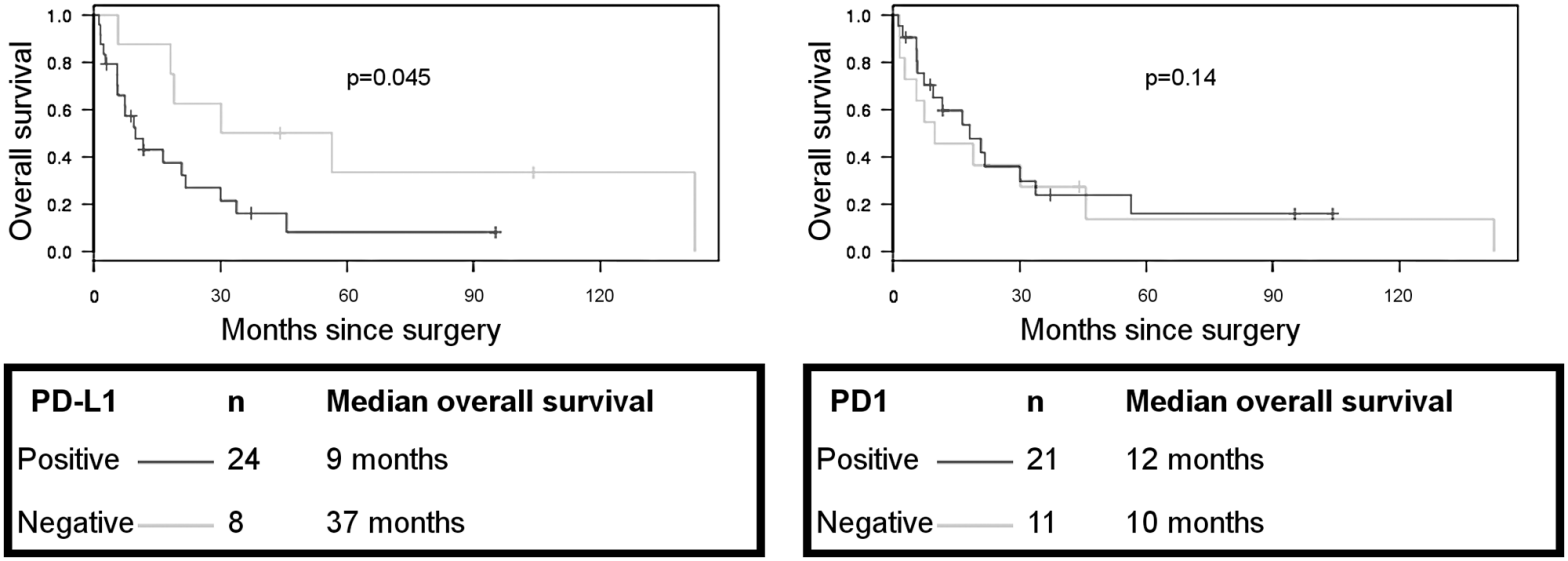
Overall survival, Kaplan-Meier curves according to PD-L1 and PD1 expression.

Four patients were excluded of survival analysis: 2 patients were lost to follow-up, 1 was diagnosed at autopsy, and 1 had a short post-operative survival (19 days). Patients with PSCGCC with an expression of PD-L1 had a worse prognosis than patients with PSCGCC without PD-L1 expression (mean = 9 vs 37 months) (p = 0.045, Fig 2). No difference in PD-L1 expression was found according to the tumor stage in the forthcoming.

PD1 expression alone is not associated with overall survival (p = 0.14, Fig 2) so as PD1 and PD-L1 coexpression (p = 0.91).

### Comparison with published studies

A synoptic comparison with published studies about PD-L1 expression in SC is provided on Table 2.

**Table 2:** Published studies on PD-L1 in sarcomatoid carcinoma of the lung.

	Number of cases	Cancer types	Use of tissue micro-array or full slides	PD-L1 clone	PD-L1 scoring	Main findings
**Velchetti et al. (2013)**	13	10 giant cell carcinoma—1 carcinosarcoma—1 pleomorphic carcinoma	Tissue microarray	5H1	Automated quantitative fluorescence analysis scoring	9 of 13 (69.2%) patients are positive compared to 122 of 445 (27.4%) conventional NSCLC
**Kim et al. (2015)**	41	Pleomorphic carcinoma	Tissue microarray	E1L3N	Scoring of intensity and percentage of tumor cells	90.2% express PD-L1. PD-L1 expression in pulmonary PCs is higher in sarcomatous areas than in the carcinomatous portion
**Gounant et al. (2016)**	1	1 SC	Not specified, presumably full slides	E1L3N	Not specified, 80% of tumor cells stained	Nivolumab-induced organizing pneumonitis
**Chang et al. (2016)**	122	Pulmonary pleomorphic carcinomas	Not specified, presumably full slides	CD274	5% on tumor cells	70.5% express PD-L1 High PD-L1 expression was significantly correlated with that of HIF-1 α and tumour necrosis. High PD-L1 expression was correlated with poor overall survival
**Vieira et al. (2016)**	75	9 carcinosarcoma; 69 PSCGC	Tissue microarray	B7H1	5% on tumor cells	53% express PD-L1. KRAS mutations, blood vessel invasion, and TTF1+ positivity were associated with PDL1 expression. Only macrophages and blood-vessel invasion were associated with PD-L1 expression on multivariate analysis
**Yvorel et al (2017)**	36	PSCGC	Full slide	E1L3N	5% on tumor cells	75% express PD-L1. PD-L1 expression is associated with TTF-1 and p40 immunohistochemistry. PD-L1 expression is correlated with a shorter overall survival

## Discussion

SC is thought to be more resistant than other NSCLC to chemotherapy and radiotherapy. A subset of SC harbors targetable mutations such as EGFR mutations, ALK or ROS 1 rearrangement. Immunotherapy targeting immune-checkpoint such as PD1/PD-L1 inhibitors might be of interest in this rare NSCLC subtype. PD-L1 expression in our study is high: from 53 to 90.2% as described in other studies [3,6–10]. PD-L1 expression in SC was found higher than in NSCLC in the same center with the same antibody and cutoff [3]. In NSCLC, PD-L1 immunohistochemistry has emerged as a biomarker predicting which patients are more likely to respond to PD/PD-L1 blockade. The best clone to use or which threshold for positivity are still subject to discussions, and some differences between studies might be explained by the different protocols used [11].

Immunohistochemistry against p40 is a very sensitive marker of squamous cell carcinoma [12]. In our work, only 8.3% of p40 positive PSCGCC expressed PD-L1.

TTF-1 and/or Napsin A stain lung adenocarcinoma whereas squamous cell carcinoma are not stained these antibodies [13]. Furthermore, in our study, 47.2% of TTF-1 and/or Napsin A positive PSCGC carcinoma were positive for PD-L1 whereas only 27.8% of TTF-1and/or Napsin A negative were positive for PD-L1. A recent study on SC found that TTF-1 positivity is associated with PD-L1 expression [3]. PD-L1 expression in PSCGC carcinoma seems to be different than other NSCLC where PD-L1 is more frequently expressed in squamous cell carcinoma than in adenocarcinoma [10].

In NSCLC, PD-L1 implication in prognosis is discordant: either indicative of an increased survival in squamous cell carcinoma [14], either indicative of a worse prognosis [15], or of no prognostic implication [16]. Our study finds a shorter overall survival in PSCGC carcinoma which is in accordance with another study in this rare subtype, nervertheless this results in our study should be interpreted with caution because the stage of patients in PD-L1 positive group is more progressive than that of PD-L1 negative group [6].

In lung adenocarcinoma, the presence of KRAS or the absence of targetable mutation are associated with PD-L1 expression, whereas EGFR or other common mutated adenocarcinoma rarely express PD-L1 [17]. A study in SC found that KRAS mutations are associated with PD-L1 expression, probably because these mutations are more likely to occur in smokers [3].

PD-L1 expression is heterogeneous in lung tumors and the use of micro-arrays may lead to false negative or positive [5]. In a study, discordant results between different cores from the same tumor was found [18]. The amount of available tissue might be a limitation to the use of micro-array for PD-L1 study, it has been proved on matched biopsies and resection specimen of NSCLC that PD-L1 expression was lower on biopsies [19]. Our study uses full tumor slides for PD1 and PD-L1 expression in order to decrease the rate of false negative results.

PD1 is the receptor binding to PD-L1. A study on pleomorphic carcinoma found that the amount of CD8+ or PD-1+ TILs and the ratio of PD-1+/CD8+ TILs in PC were higher in males, smokers and older patients. In our study, PD1 was not related to any of the parameters studied neither to PD-L1 expression. Nevertheless, PD1 expression is not correlated to immunotherapy response.

The main limitation of our study is the number of patients, but PSCGC is a rare tumor type among lung neoplasms [4]. The other limitation is the about which PD-L1 antibody clone is able to predict response to immunotherapy. This question and the reproductibility of testing with different antibody clones is still debated [11].

Our work confirms the high rate of PD-L1 expression in PSCGCC, which represents a hope for these patients which are more resistant to current therapies. Nevertheless, clinical response in this specific cancer type has not been specifically evaluated. In contrast with NSCLC, PD-L1 expression in PSCGCC is related to the presence of adenocarcinoma markers such as TTF-1 and/or Napsin A. Another hope for patients with lung SC is the relatively higher frequency in this subtype of exon 14 skipping of MET (hepatocyte growth factor receptor gene) [20]. This is a molecular alteration which might be targetable by mesenchymal-to-epithelial transition factor tyrosine kinase inhibitors [20].These findings with ours are of interest because PSCGCC seems different from NSCLC in their PD-L1 expression and gene expression profile, this might be a help for patient selection for PD-L1 testing and molecular testing.
